# Single-cell eQTL Mendelian randomization reveals the monocyte-associated PRKCB–CTSL axis as a candidate regulatory pathway and potential therapeutic target in rheumatoid arthritis

**DOI:** 10.1007/s10067-026-08482-4

**Published:** 2026-10-19

**Authors:** Pengli Wang, Guohang Shen, Wenhua Liao, Ruoyan Wang, Li Zhang, Mingjia Zhao, Dong Zhang, Nanxiang Huang, Yupei Dai

**Affiliations:** 1 Department of Pediatric Surgery, Beijing Anzhen Nanchong Hospital of Capital Medical University & Nanchong Central Hospital, Second Clinical Medical College of North Sichuan Medical College, No. 97 Renmin South Road, Shunqing, Nanchong, 637000 Sichuan China; 2 School of Basic Medicine and Forensic Medicine, North Sichuan Medical College, No.234 Fujiang Road, Shunqing District, Nanchong , Sichuan, 637000 China; 3 North Sichuan Medical College, No. 234 Fu Jiang Road, Shunqing, Nanchong, 637000 Sichuan People’s Republic of China; 4 Department of Clinical Medicine, Ningxia Medical University, Hui Autonomous Region, Yinchuan, Ningxia 750004 People’s Republic of China

**Keywords:** Mendelian randomization, Molecular docking, Rheumatoid arthritis, Single-cell eQTL, Single-cell transcriptomics

## Abstract

**Objectives:**

Rheumatoid arthritis (RA) involves persistent immune dysregulation, but cell-specific genetically regulated pathways remain unclear. We integrated cell-specific genetic and multi-omics evidence to prioritize candidate regulatory pathways.

**Methods:**

cis-eQTLs from 17 immune-cell populations were integrated with RA GWAS data by two-sample Mendelian randomization. Transcriptomics, WGCNA, and machine-learning feature selection identified convergent candidates. Single-cell and virtual perturbation analyses assessed cellular localization and downstream changes. Molecular docking and dynamics computationally prioritized compounds, and knockdown/rescue experiments evaluated the PRKCB–CTSL relationship.

**Results:**

Eleven candidates had convergent genetic and transcriptomic support. PRKCB was retained by LASSO, SVM, and random forest and preferentially expressed in monocytes. Virtual perturbation implicated CTSL and antigen-processing/presentation pathways. PRKCB knockdown reduced CTSL expression and NF-κB phosphorylation, whereas CTSL restoration partially rescued NF-κB phosphorylation. Separately, structure-based analyses computationally prioritized ursodiol for the most favorable predicted PRKCB interaction among evaluated compounds.

**Conclusions:**

Genetic, transcriptomic, single-cell, computational, and in vitro evidence supports monocyte-associated PRKCB–CTSL as a candidate regulatory pathway in RA. Further mechanistic and in vivo studies are required to establish its causal and pharmacological relevance.

**Table:** 

**Key Points** • *Multi-omics analysis identified 11 key RA-associated targets.* • *RA was classified into two molecular subtypes with distinct immune features.* • *PRKCB was identified as the core target enriched in monocytes.* • *The PRKCB–CTSL axis may drive RA inflammation, and ursodiol showed therapeutic potential.*

**Supplementary Information:**

The online version contains supplementary material available at https://doi.org/10.1007/s10067-026-08482-4.

## Introduction

Rheumatoid arthritis (RA) is a chronic autoimmune disorder characterized by persistent synovial inflammation, progressive cartilage and bone destruction, and systemic immune dysregulation, ultimately leading to irreversible joint deformity and multi-organ impairment. The disease imposes a substantial burden on both patient quality of life and global healthcare systems [1–4]. The pathogenesis of RA is highly heterogeneous and involves complex interactions among genetic susceptibility, aberrant immune-cell activation, dysregulated transcriptional programs, and inflammatory signaling networks. Among these factors, immune cell-specific transcriptional regulation driven by genetic variation has emerged as a critical mechanism underlying RA initiation and progression [5–7]. Although disease-modifying antirheumatic drugs (DMARDs) and biologic therapies have significantly improved clinical outcomes, considerable challenges remain, including heterogeneous therapeutic responses, limited target specificity, and long-term adverse effects associated with chronic treatment [8, 9]. Therefore, systematic identification of causally relevant pathogenic genes through integrated genetic and transcriptomic analyses is essential for the development of precision molecular stratification and targeted therapeutic strategies in RA.

Recent advances in bulk and single-cell omics have substantially expanded the molecular landscape of RA, revealing disease-associated transcriptional programs, immune-cell heterogeneity, and dysregulated inflammatory networks. Bulk transcriptomic studies are particularly valuable for identifying reproducible disease-associated signatures and co-expression modules across patient cohorts; however, signals measured at the tissue level may reflect changes in cellular composition and cannot readily resolve the immune-cell context in which genetically regulated transcriptional effects arise. Conversely, single-cell RNA sequencing enables high-resolution characterization of cellular states and lineage-specific expression patterns but, when used alone, remains primarily observational and does not directly establish whether cell-specific transcriptional differences are linked to inherited genetic regulation of RA susceptibility [10, 11]. Thus, an important unresolved challenge is to bridge disease-associated transcriptional changes with cell-type-specific genetic regulation and subsequently determine whether genetically prioritized signals converge with independent transcriptomic and functional evidence.

Single cell eQTL analysis provides an opportunity to address this gap by mapping the effects of genetic variants on gene expression within defined cellular populations. When integrated with RA GWAS data, cell-type-specific cis-eQTLs can therefore prioritize gene-expression traits whose genetically predicted variation is associated with disease susceptibility in a defined immune-cell context [12–14]. This framework is conceptually distinct from conventional differential-expression analyses because it moves the analytical starting point from disease-associated expression changes toward genetically anchored, cell-resolved regulatory effects. Nevertheless, genetic prioritization alone cannot establish the cellular localization, downstream regulatory consequences, or functional relevance of a candidate gene. Integration with independent transcriptomic cohorts, co-expression networks, single-cell expression profiles, computational perturbation, and experimental validation is therefore required to construct a more comprehensive evidence chain.

Beyond immune-cell composition and genetic regulation, cellular stress-response programs represent an additional layer of biological complexity relevant to chronic inflammatory disease. Recent transcriptomic profiling has demonstrated that pharmacological stress can induce coordinated remodeling of DNA-repair and autophagy programs, illustrating the capacity of transcriptome-wide approaches to resolve interconnected adaptive responses to cellular perturbation [15]. In parallel, studies of plant-derived selenium nanoparticles and Prosopis cineraria seed extracts have highlighted the biological activities of redox-modulating and antioxidant strategies in experimental systems [16, 17]. Although these studies were conducted outside the context of RA and do not directly establish a relationship with PRKCB–CTSL signaling, they underscore the broader importance of integrating stress-responsive molecular networks with disease-specific genetic and cellular evidence when identifying candidate therapeutic pathways.

Against this background, we integrated cell-type-specific cis-eQTLs from 17 immune-cell populations with RA GWAS data to genetically prioritize RA-associated eGenes, followed by transcriptomic, WGCNA, and machine-learning analyses to identify key targets. Single-cell transcriptomics and virtual gene perturbation were further used to resolve cellular specificity and potential regulatory relationships. Finally, drug-screening analyses and in vitro validation were performed to explore translational relevance. This integrated framework links cell-type-specific genetic evidence with multi-omics and experimental validation to identify candidate regulatory pathways and therapeutic targets in RA.

## Materials and methods

### Two-sample Mendelian randomization analysis

Cell-type-specific cis-expression quantitative trait loci (cis-eQTLs) derived from 17 immune cell populations were used as genetic instruments for gene expression, and RA GWAS summary statistics were used as the outcome. Each exposure was defined at the cell-type–gene level; thus, the MR estimate represented the association between genetically predicted expression of a specific eGene within a specific immune-cell population and RA susceptibility.

Instrumental variants were restricted to genome-wide significant cis-eQTLs (P < 5 × 10⁻⁸) and were clumped to retain independent variants (r^2^ < 0.001; clumping window > 1,000 kb). Because cell-type-specific cis-eQTL mapping frequently yielded only one eligible independent instrumental variant for a given cell-type–gene pair after significance filtering and LD clumping, an instrument-number-dependent MR strategy was adopted. For exposures represented by a single eligible cis-eQTL, the MR effect was estimated using the Wald ratio, calculated as β_GY/β_GX, where β_GY denotes the genetic association with RA and β_GX denotes the genetic association with expression of the corresponding eGene in the specified immune-cell population. The standard error of the ratio estimate was derived using the delta method.

For cell-type–gene exposures with multiple eligible independent cis-eQTL instruments, the inverse-variance weighted (IVW) method was used to combine SNP-specific Wald estimates. Where the number of available instruments permitted reliable implementation, complementary multi-instrument analyses, including MR-Egger regression and weighted-median estimation, were performed to evaluate robustness to alternative assumptions. Pleiotropy-oriented sensitivity analyses were likewise restricted to exposure–instrument sets for which their statistical requirements were satisfied, because regression-, median-, and outlier-based procedures cannot be meaningfully applied to single-instrument MR.

Importantly, the use of cis-eQTL instruments was intended to increase the biological specificity of the genetic proxy for gene expression but was not assumed to eliminate horizontal pleiotropy. In particular, for single-instrument Wald-ratio estimates, horizontal pleiotropy cannot be empirically assessed using multi-instrument methods such as MR-Egger or MR-PRESSO. These estimates were therefore interpreted as cell-type-specific genetic evidence consistent with a potentially causal association, rather than definitive proof of causality. Potential bias related to sample overlap and weak-instrument effects was additionally evaluated using MRlap where applicable [12, 18].

### Differential expression analysis and WGCNA

RA-related transcriptomic datasets were downloaded from the Gene Expression Omnibus (GEO) database. Batch effects were corrected using the ComBat algorithm implemented in the sva package. Differential expression analysis was subsequently performed using the limma package, with |log₂FC|> 0.5 and adjusted *P* < 0.05 (FDR) set as the screening criteria. Differentially expressed genes (DEGs) between RA and control samples were identified and categorized into upregulated and downregulated gene sets [19, 20].

Weighted gene co-expression network analysis was performed using the WGCNA R package. Expression data were normalized using normalizeBetweenArrays, and genes with a standard deviation > 0.5 across samples were retained for network construction. Sample and gene quality was assessed using goodSamplesGenes, followed by average-linkage hierarchical clustering to identify potential outlier samples. Candidate soft-thresholding powers ranging from 1 to 20 were evaluated using pickSoftThreshold, and the estimated optimal power was used to construct the adjacency matrix. The adjacency matrix was subsequently transformed into a topological overlap matrix (TOM), followed by average-linkage hierarchical clustering based on TOM dissimilarity. Gene modules were identified using dynamic tree cutting with a minimum module size of 50, deepSplit = 2, and pamRespectsDendro = FALSE. Highly similar modules were subsequently merged using a module eigengene dissimilarity threshold of 0.25. Pearson correlations between module eigengenes and RA status were calculated to identify disease-associated modules for subsequent integrative analyses [21, 22].

### Identification of key RA-associated targets

Risk genes with causal effect estimates > 1 identified by two-sample MR analysis were intersected with upregulated differentially expressed genes from GEO transcriptomic datasets and genes within disease-positive WGCNA core modules. Similarly, protective genes with causal effect estimates < 1 were intersected with downregulated differentially expressed genes and genes from disease-negative core modules. Genes identified from both intersection analyses were defined as key RA-associated targets.

### Functional enrichment and protein–protein interaction (PPI) analysis of key targets

Gene Ontology (GO) functional enrichment and Kyoto Encyclopedia of Genes and Genomes (KEGG) pathway enrichment analyses were performed using the clusterProfiler and org.Hs.eg.db packages in R. GO analysis included biological process (BP), cellular component (CC), and molecular function (MF) categories. The identified key targets were subsequently submitted to the GeneMANIA online platform (https://genemania.org/) for PPI network construction [23, 24].

### Identification and validation of RA molecular subtypes

Consensus clustering was performed based on the normalized expression matrix of key targets to classify RA molecular subtypes. The ConsensusClusterPlus package was used for clustering analysis, and the optimal cluster number was determined according to cumulative distribution function (CDF) curves and consensus matrices. Principal component analysis (PCA) was subsequently conducted using the FactoMineR, factoextra, and ggplot2 packages to evaluate the separation of expression patterns among different subtypes.

Single-sample gene set enrichment analysis (ssGSEA) was performed using the GSVA and GSEABase packages to quantify immune cell infiltration based on immune cell signature gene sets, followed by comparison of immune infiltration characteristics between subtypes. GSVA was further applied to calculate KEGG pathway enrichment scores and identify significantly altered signaling pathways among different subtypes [25].

### Machine learning-based identification of core targets

Least absolute shrinkage and selection operator (LASSO) regression, support vector machine (SVM), and random forest (RF) algorithms were jointly applied to identify core pathogenic targets in RA. LASSO regression was performed using the glmnet package (v4.1–6), with disease phenotype as the dependent variable and key target expression profiles as independent variables. Ten-fold cross-validation was conducted to determine the optimal λ value, and genes with non-zero regression coefficients were retained as candidate features. SVM analysis was implemented using the e1071 package (v1.7–13) with a radial basis function kernel, and model parameters were optimized through cross-validation. Candidate genes were selected according to feature weights. RF analysis was conducted using the randomForest package (v4.7–1.1), with the number of decision trees set to 500, and gene importance was evaluated based on Mean Decrease Accuracy scores [26]. Genes consistently identified by all three algorithms were defined as core RA-associated targets.

### Immune infiltration and single-gene GSEA analysis

Single-sample gene set enrichment analysis was performed to quantify immune cell infiltration across all samples. Pearson correlation coefficients between the core target and immune cell infiltration levels were calculated, and correlation heatmaps were generated using the corrplot package.

Gene set enrichment analysis (GSEA) was subsequently performed using the clusterProfiler package, with core target expression treated as a continuous phenotype variable. The reference gene set “c2.cp.kegg.v7.5.1.symbols.gmt” was used to identify KEGG signaling pathways significantly associated with core target expression.

### Single-cell transcriptomic analysis

RA-related single-cell transcriptomic datasets were downloaded from the GEO database for cellular localization analysis of the core target. Single-cell transcriptomic data were processed using Seurat. Genes detected in fewer than three cells were excluded during object construction, and cells with fewer than 200 or at least 5000 detected genes or with ≥ 20% mitochondrial transcripts were removed. Gene-expression counts were normalized using the LogNormalize method with a scale factor of 10,000, and 1500 highly variable genes were identified using the “vst” method. Following data scaling, principal component analysis was performed using 50 principal components. Batch effects across samples were subsequently corrected using Harmony with sample identity (orig.ident) as the grouping variable. The first 30 Harmony dimensions were used to construct the nearest-neighbor graph, and unsupervised clustering was performed using FindClusters with a resolution of 0.5 and algorithm 1. UMAP was used for visualization, and cell identities were assigned according to established canonical marker genes. For monocyte-focused analysis, the Harmony-corrected object was further subclustered using 29 dimensions and a clustering resolution of 0.3, followed by marker-based annotation of monocyte subpopulations.

### scTenifoldKnk-based virtual gene-knockout analysis

To investigate transcriptional changes potentially associated with perturbation of PRKCB, virtual gene-knockout analysis was performed using the scTenifoldKnk R package. Raw count data were extracted from the Seurat object, and 10,000 highly variable genes were identified using the “vst” method. The target gene was retained together with the highly variable genes to construct the input expression matrix. Virtual perturbation was performed with quality control enabled (qc = TRUE), a mitochondrial-content threshold of 0.1 (qc_mtThreshold = 0.1), a minimum library-size threshold of 1000 (qc_minLSize = 1000), 10 independently constructed subnetworks (nc_nNet = 10), and 500 randomly sampled cells per subnetwork (nc_nCells = 500). A fixed random seed (123) was used to improve computational reproducibility. Genes showing significant regulatory changes following virtual perturbation were identified at an adjusted *P* value < 0.05 and were subsequently evaluated as candidate genes associated with PRKCB perturbation. GO and KEGG enrichment analyses were subsequently performed to evaluate biological pathways potentially regulated following core target knockout [27].

### Candidate drug screening and molecular docking

Small-molecule compounds targeting the core gene were screened using the DSigDB database. Compounds with reported toxic effects were excluded, and FDA-approved drugs were retained for subsequent analyses.

The two-dimensional structures of candidate compounds were obtained from PubChem and converted into optimized three-dimensional conformations using Chem3D 23.1.1. A high-resolution crystal structure of PRKCB was retrieved from the RCSB Protein Data Bank. Water molecules, phosphate groups, and other nonessential heteroatoms were removed using PyMOL 2.6. Receptor and ligand structures were subsequently prepared using AutoDockTools, including hydrogen addition, charge assignment, and definition of ligand rotatable bonds. Molecular docking was performed using AutoDock Vina 1.2.5. Candidate binding poses were ranked according to their Vina docking scores, with more negative scores representing more favorable predicted interactions within the docking model. Selected protein–ligand interaction patterns were visualized using Discovery Studio 2019 and PyMOL 2.6.

### Molecular dynamics simulation

Molecular dynamics (MD) simulations of the protein–ligand complex were conducted using GROMACS. The protein was parameterized with the Amber99SB-ILDN force field, whereas ligand parameters were generated using sobtop_1.0 (dev3.1) under the GAFF2 framework. RESP-derived atomic charges were assigned to the ligand to represent its electrostatic properties. The complex was placed at the center of a cubic periodic box with a minimum solute-to-boundary distance of 1.0 nm and solvated explicitly using the TIP3P water model. Na⁺ and Cl⁻ ions were introduced with gmx genion to neutralize the system and establish a final NaCl concentration of 0.15 M. Long-range electrostatic interactions were treated using the particle mesh Ewald (PME) scheme, while a cutoff of 1.2 nm was applied to short-range electrostatic and van der Waals interactions. Covalent bonds were constrained using the LINCS algorithm, allowing an integration time step of 2 fs. Prior to equilibration, unfavorable contacts and high-energy local geometries were relaxed through sequential steepest-descent and conjugate-gradient energy minimization. The minimized system was then equilibrated for 1 ns under the NVT ensemble followed by 1 ns under the NPT ensemble, with positional restraints applied to both the protein and ligand. Temperature was maintained at 300 K using the velocity-rescaling thermostat with a coupling constant of 1 ps. During NPT equilibration, the pressure was regulated at 1 bar using the Parrinello–Rahman barostat with a pressure-coupling constant of 1 ps. After equilibration, the positional restraints were removed for the production MD simulation. Trajectory analyses were subsequently performed using GROMACS utilities. Global conformational stability was examined from backbone root mean square deviation (RMSD), whereas residue-level mobility was characterized by root mean square fluctuation (RMSF). The radius of gyration (Rg) and solvent-accessible surface area (SASA) were calculated to monitor changes in structural compactness and solvent exposure, respectively. Protein–ligand hydrogen bonds were quantified throughout the trajectory to characterize the persistence of intermolecular contacts. Principal component analysis (PCA) was further combined with gmx sham to reconstruct the free-energy landscape (FEL) and identify energetically preferred conformational states. Finally, the binding free energy of the protein–ligand complex was estimated using gmx_MMPBSA within the MM/PBSA framework, providing an energetic assessment complementary to the structural and dynamic analyses.

### Drug safety assessment

Adverse event (AE) reports from the FDA Adverse Event Reporting System (FAERS) database between the first quarter of 2004 and the second quarter of 2025 were retrieved for safety evaluation. Data preprocessing was performed using the tidyverse, readr, and dplyr packages in R, including duplicate removal, MedDRA terminology standardization, and selection of reports in which the target drug was identified as the primary suspected agent. Eligible AE reports associated with the target drug were subsequently included for analysis.

The analyses included demographic characteristics (age and sex distribution), system organ class (SOC) involvement, and high-frequency adverse events. Multivariable logistic regression models were constructed using the stats package to adjust for potential confounding factors, including age, body weight, and off-label drug use. Odds ratios (ORs) and 95% confidence intervals (CIs) were calculated, and *P* < 0.05 was considered statistically significant for identifying independent risk factors.

### In vitro experimental validation

To investigate the functional role of PRKCB in the inflammatory response associated with rheumatoid arthritis (RA) and its potential regulatory effects on downstream CTSL and NF-κB signaling, human monocytes were used for in vitro experiments. An RA-related inflammatory cell model was established by TNF-α stimulation, and two independent experimental settings were designed for functional validation.

In the first experiment, PRKCB knockdown was performed using four groups: the normal control group (control), RA model group (disease), negative-control siRNA-transfected group (disease + si-NC), and PRKCB knockdown group (disease + si-PRKCB). PRKCB-specific small interfering RNA (si-PRKCB) was transfected into cells using Lipofectamine™ 3000 according to the manufacturer’s instructions. A nonspecific negative-control siRNA (si-NC) was transfected in parallel to control for potential nonspecific effects associated with the transfection procedure.

In the second experiment, a CTSL rescue assay was performed using four groups: control, disease, disease + si-PRKCB, and PRKCB knockdown combined with CTSL overexpression (disease + si-PRKCB + OE-CTSL). In the rescue group, a CTSL overexpression plasmid (OE-CTSL) was co-transfected in the context of PRKCB knockdown to determine whether restoration of CTSL expression could reverse the downstream molecular changes induced by PRKCB depletion, thereby providing functional evidence for the proposed regulatory relationship between PRKCB and CTSL.

Following the indicated treatments, total protein was extracted using RIPA lysis buffer supplemented with protease and phosphatase inhibitors, and protein concentrations were determined using the bicinchoninic acid (BCA) assay. Equal amounts of protein were separated by SDS-PAGE and transferred onto polyvinylidene fluoride (PVDF) membranes. The membranes were blocked with 5% nonfat milk for 1 h at room temperature and subsequently incubated overnight at 4 °C with primary antibodies against PRKCB, CTSL, phosphorylated NF-κB (p-NF-κB), total NF-κB, and β-actin. After washing, the membranes were incubated with the corresponding secondary antibodies for 1 h at room temperature, and protein bands were visualized using enhanced chemiluminescence (ECL). Band intensities were quantified using ImageJ, and relative protein expression levels were normalized to β-actin.

All experiments were independently repeated at least three times. Data are presented as the mean ± standard deviation (SD). Comparisons among multiple groups were performed using one-way analysis of variance (ANOVA), followed by Tukey’s post hoc test. A two-sided *P* < 0.05 was considered statistically significant.

## Results

### Two-sample MR analysis of immune cell-specific eGenes associated with RA risk

Two-sample MR analysis was performed using eGenes from 17 immune cell populations as exposure variables and RA GWAS summary statistics as outcome data. A total of 842 immune cell-specific eGenes were identified as significantly associated with RA susceptibility. Among them, 451 genes exhibited causal effect estimates > 1 and were classified as risk genes, whereas 391 genes with effect estimates < 1 were classified as protective genes, providing the candidate gene set for subsequent multi-omics integration analyses. Detailed information is provided in Supplementary Table 1.

### Differential expression analysis and WGCNA in RA

**Fig. 1:**
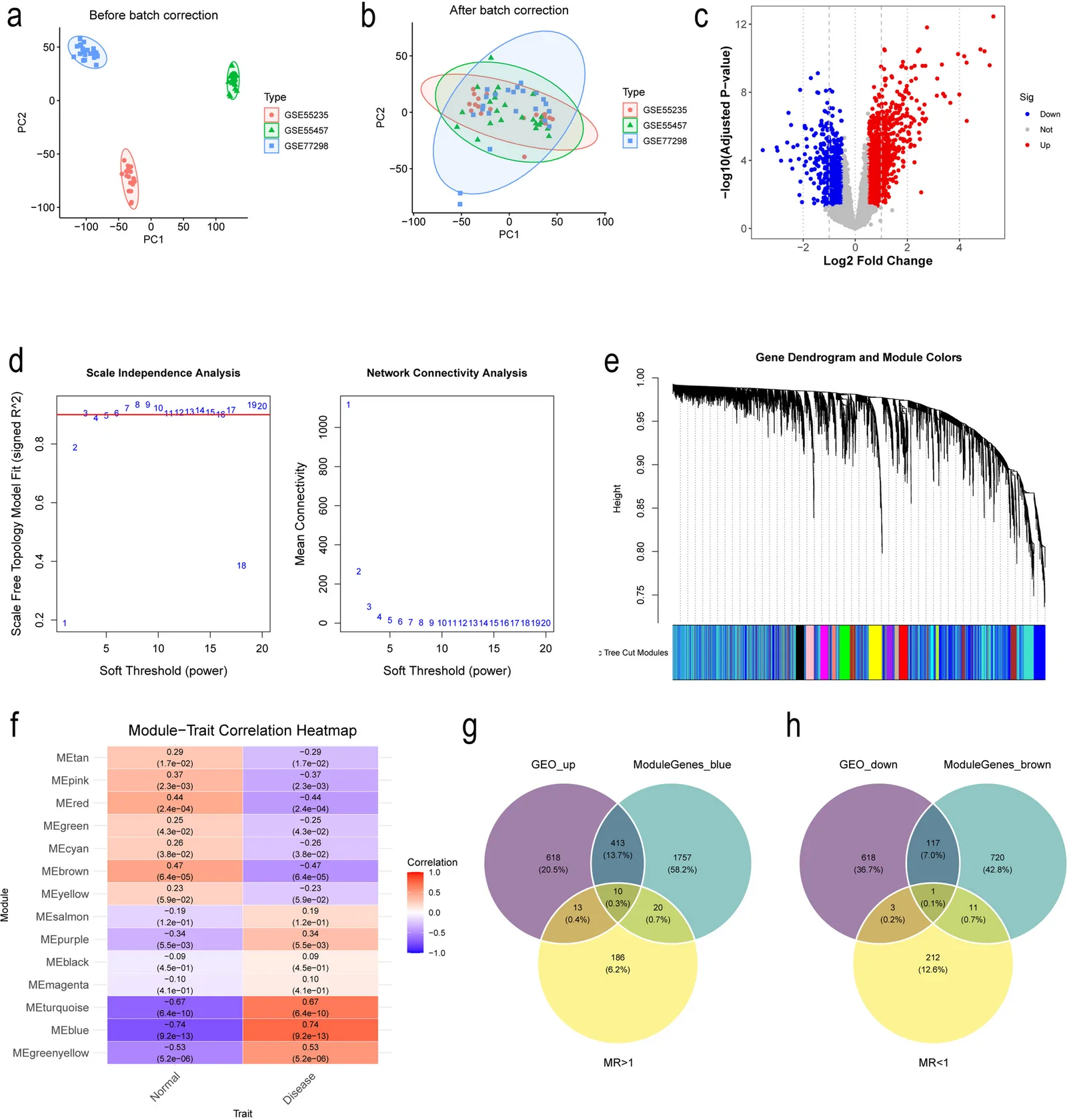
Integrated transcriptomic analysis and identification of key targets in RA. **a**–**b** PCA before and after batch-effect correction. **a** Samples from the GSE55235, GSE55457, and GSE77298 datasets showed obvious batch separation before correction. **b** Sample distributions became more consistent after correction, indicating effective removal of batch effects. **c** Volcano plot of DEGs between RA and healthy control samples. **d** Soft-threshold selection for WGCNA. **e** Hierarchical clustering dendrogram and co-expression module identification. **f** Module–trait relationship heatmap showing correlations between co-expression modules and disease status. **g**–**h** Venn diagrams for identification of key targets. **g** Intersection of upregulated DEGs, genes from the WGCNA blue module, and MR risk genes (MR > 1). **h** Intersection of downregulated DEGs, genes from the WGCNA brown module, and MR protective genes (MR < 1). Overlapping genes were defined as candidate key targets for RA

Three RA-related transcriptomic datasets, including GSE55235, GSE55457, and GSE77298, were obtained from the GEO database. Following dataset integration, batch effect correction, and differential expression analysis, a total of 1795 DEGs were identified, including 1055 upregulated and 740 downregulated genes (Fig. 1a–c; Supplementary Fig. 1a). The complete DEG list, including gene-level log2FC values and FDR-adjusted *P* values, is provided in Supplementary Table 2.

WGCNA was subsequently conducted based on the identified DEGs. A scale-free co-expression network was successfully established, and 14 co-expression modules were identified (Fig. 1d–f; Supplementary Fig. 1b). Module–trait correlation analysis demonstrated that the MEblue module was most positively correlated with RA status, whereas the MEbrown module showed the strongest negative correlation. These two modules were therefore selected as the core modules for subsequent target identification analyses. Complete gene-to-module assignments generated by WGCNA are provided in Supplementary Table 3.

### Multi-omics identification of key RA-associated targets

**Fig. 2:**
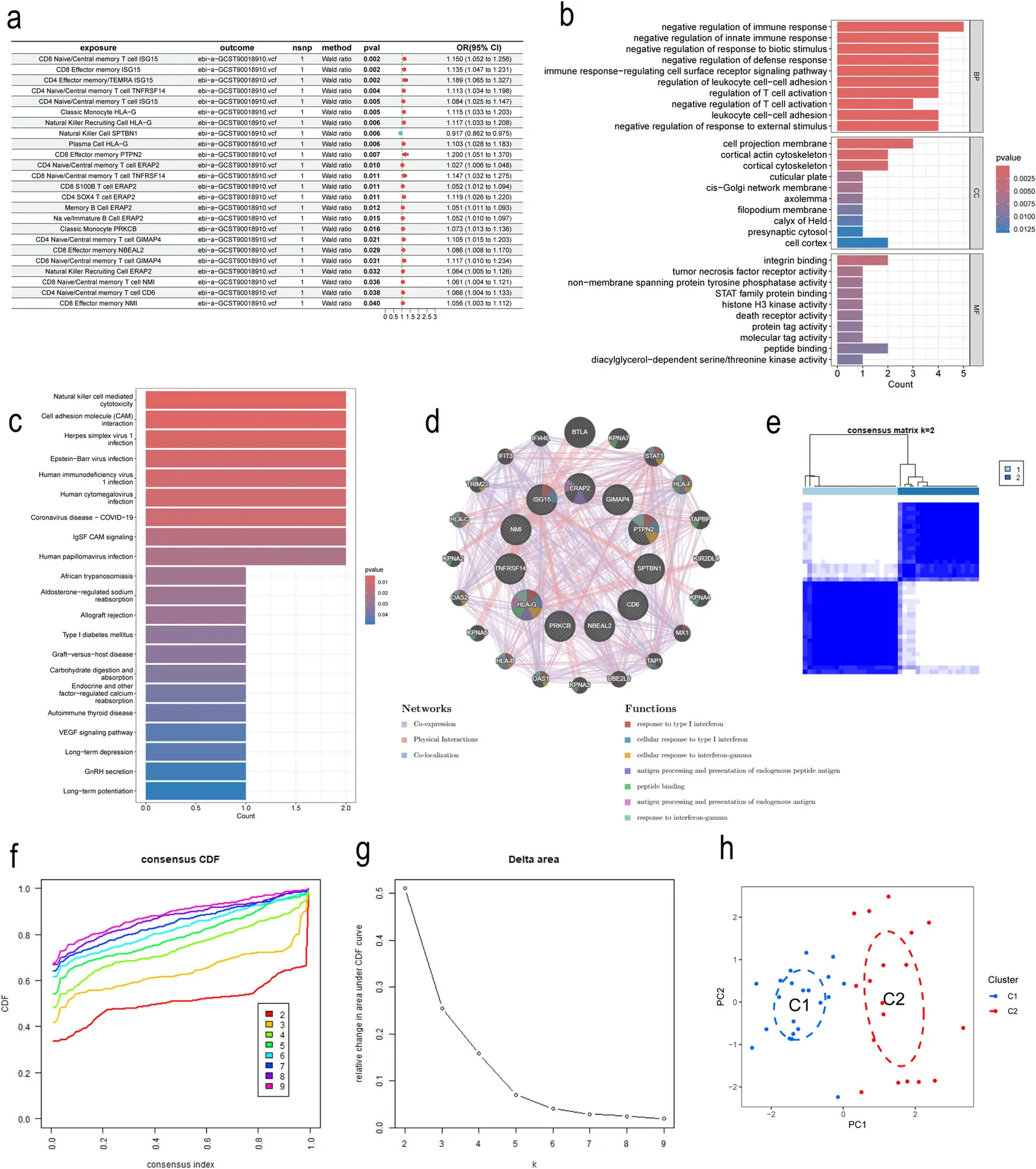
Causal gene identification, functional enrichment, and molecular subtype analysis in RA. **a** Forest plot of two-sample Mendelian randomization (MR) analysis based on single-cell eQTL data. **b** GO enrichment analysis of key genes. **c** KEGG pathway enrichment analysis of key genes. **d** PPI network of key genes. **e**–**h** Consensus clustering analysis for RA molecular subtypes. **e** Consensus matrix heatmap showing clustering consistency among samples. **f** CDF curves for different cluster numbers (*k* = 2–9). **g** Delta area plot showing relative changes in the area under the CDF curve. **h** PCA plot based on key gene expression profiles, demonstrating the distribution differences between the C1 and C2 subtypes

A multi-omics intersection strategy was applied for stepwise screening of candidate genes. genes with MR effect estimates in the risk-increasing direction (OR > 1) identified by two-sample MR analysis were intersected with upregulated differentially expressed genes from GEO datasets and genes from the positively correlated MEblue module identified by WGCNA, resulting in 10 candidate genes (Fig. 1g). In parallel, genes with MR effect estimates in the risk-decreasing direction (OR < 1) were intersected with downregulated differentially expressed genes and genes from the negatively correlated MEbrown module, yielding one additional candidate gene (Fig. 1h). After integration of both gene sets, a total of 11 key RA-associated targets were identified. Forest plots were subsequently generated to illustrate the association effects of these targets with RA (Fig. 2a).

### Functional enrichment and PPI network analysis of key targets

GO enrichment analysis of the 11 key targets demonstrated significant enrichment in pathways related to negative regulation of immune response, negative regulation of innate immune response, negative regulation of response to biotic stimulus, membrane of cellular projections, cortical actin cytoskeleton, and integrin binding across biological process, cellular component, and molecular function categories (Fig. 2b). KEGG pathway analysis further indicated enrichment in pathways associated with natural killer cell-mediated cytotoxicity, cell adhesion molecule (CAM) interactions, and Epstein–Barr virus infection (Fig. 2c). PPI network analysis based on the GeneMANIA database revealed strong interactions among the 11 key targets, suggesting their coordinated involvement in RA pathogenesis (Fig. 2d).

### Molecular subtyping of ra based on key targets

**Fig. 3:**
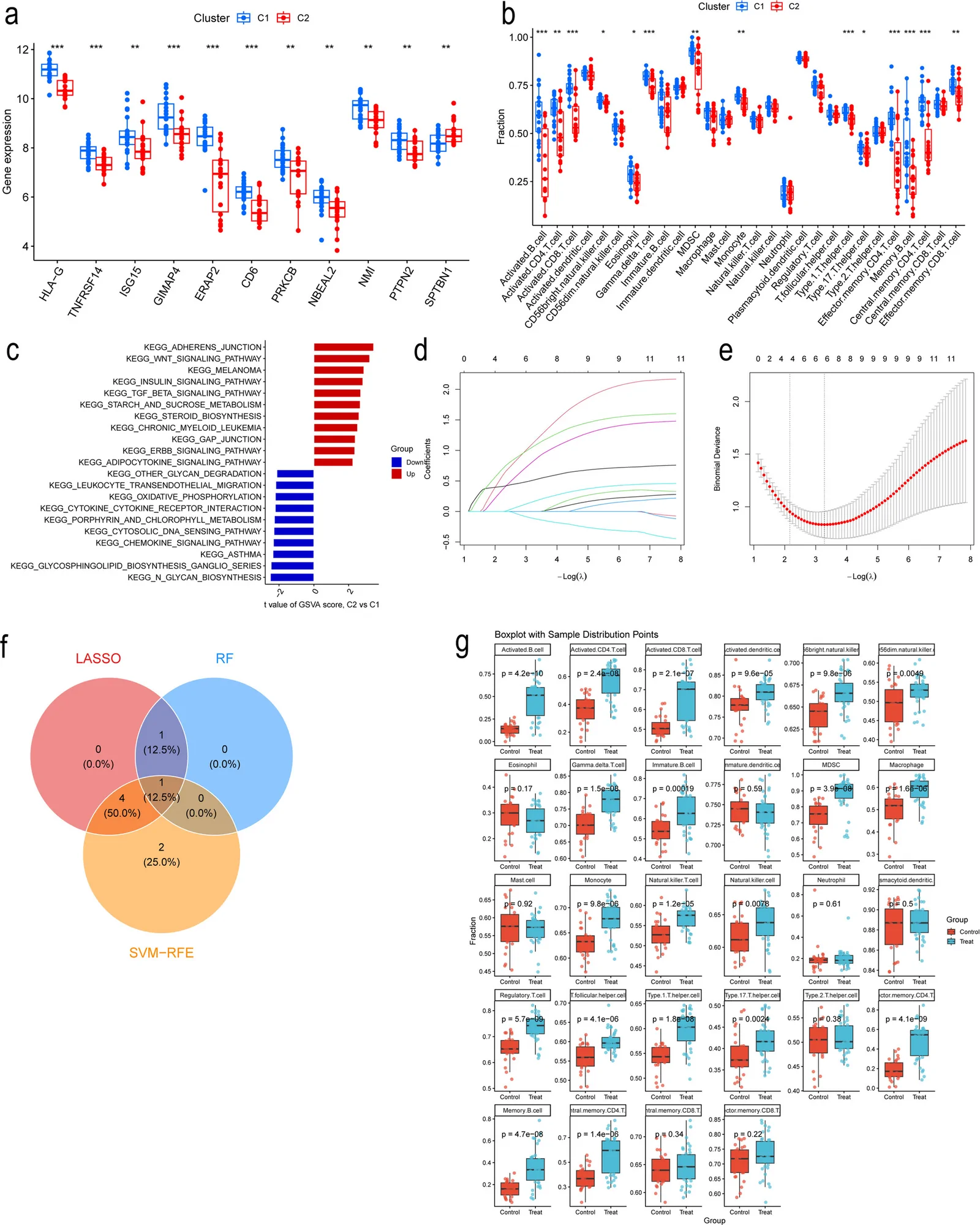
Characterization of RA molecular subtypes and identification of core pathogenic targets. **a** Boxplots showing differential expression of key candidate genes between the C1 and C2 RA subtypes. **b** Comparison of immune cell infiltration levels between the C1 and C2 subtypes. **c** GSVA of KEGG pathways across RA molecular subtypes. **d**–**e** LASSO regression analysis for identification of core pathogenic genes in RA. **d** Coefficient profiles of candidate genes under different penalty parameter (λ) values. **e** Partial likelihood deviance curve based on tenfold cross-validation, with dashed lines indicating the optimal λ value. **f** Venn diagram showing overlapping genes identified by LASSO regression, RF, and SVM-RFE, with intersecting genes defined as core candidate targets for RA. **g** Validation of differential immune cell infiltration levels between healthy controls (control) and RA samples (test)

Consensus clustering based on the 11 candidate targets identified two RA molecular subtypes, C1 and C2, which showed distinct expression patterns by PCA (Fig. 2e–h). Most candidate targets showed relatively higher expression in C1, whereas SPTBN1 was higher in C2 (Fig. 3a). Immune-profile analysis further revealed greater infiltration of several activated lymphocyte populations in C1, including activated B cells and activated/effector-memory T-cell populations (Fig. 3b). In parallel, GSVA demonstrated distinct pathway-activity patterns between the two subtypes, involving immune-inflammatory and tissue-remodeling-related processes (Fig. 3c). Collectively, these findings indicate that C1 and C2 represent transcriptionally and immunologically distinct RA molecular patterns.

### Identification of the core RA target using machine learning algorithms

LASSO regression, SVM, and RF algorithms were jointly applied to identify core targets among the 11 candidate genes. LASSO regression identified six feature genes (Fig. 3d, e), SVM analysis identified seven feature genes (Supplementary Fig. 1c), and RF analysis identified two feature genes (Supplementary Fig. 1 d, e). Intersection of the feature sets identified by LASSO, SVM, and RF revealed PRKCB as the only gene consistently selected by all three algorithms (Fig. 3f). PRKCB was therefore prioritized as the core candidate RA-associated target for subsequent cellular, mechanistic, and translational analyses.

### Immune infiltration and single-gene GSEA analysis of PRKCB

**Fig. 4:**
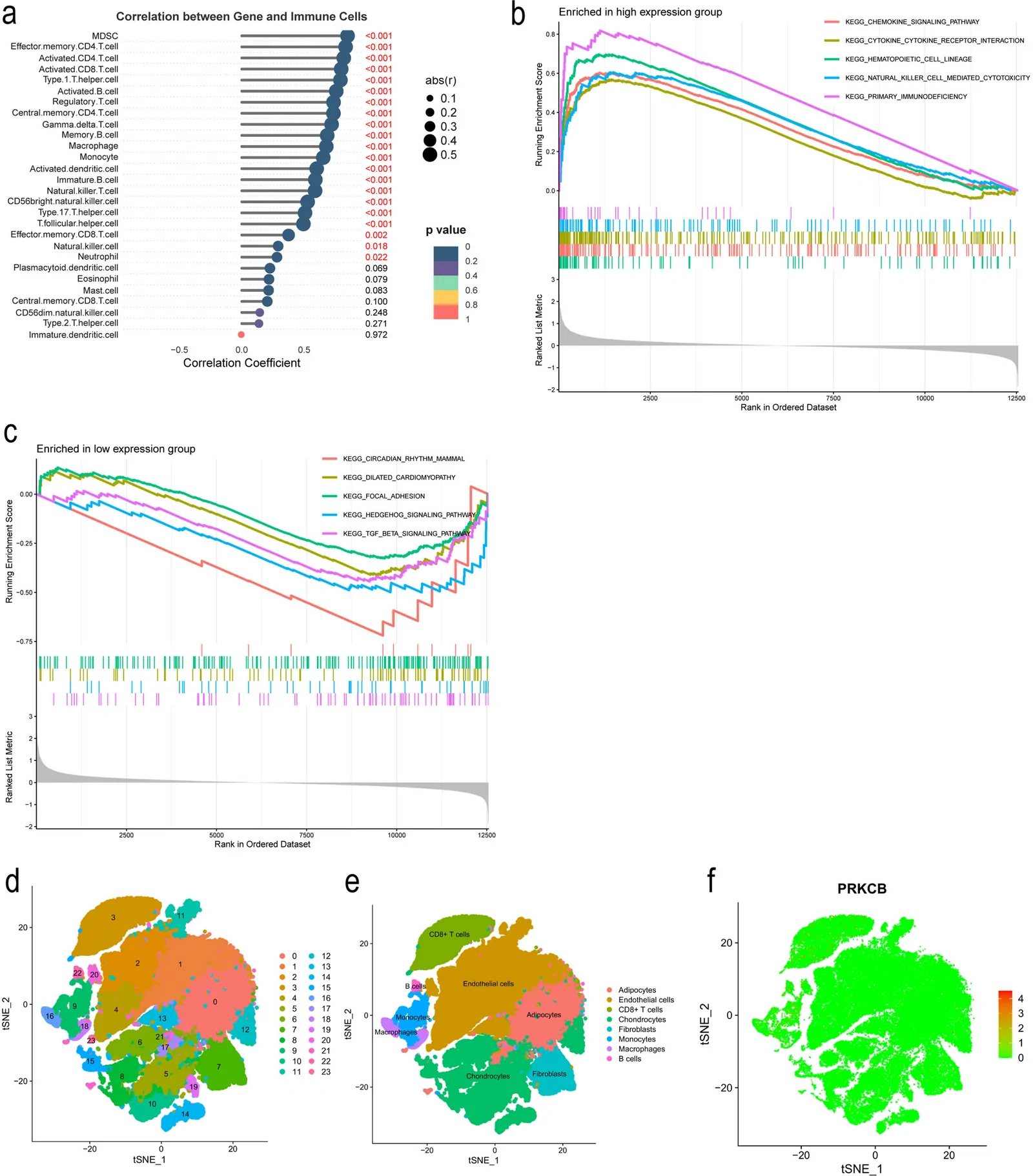
Association of the core target PRKCB with the immune microenvironment and its single-cell expression profile. **a** Correlation analysis between PRKCB expression and immune cell infiltration levels. **b** GSEA results in the PRKCB high-expression group. **c** GSEA results in the PRKCB low-expression group. **d** t-SNE plot of unsupervised clustering based on single-cell transcriptomic data from RA synovial tissue. **e** Cell-type annotation t-SNE plot of single-cell transcriptomic data, with different colors representing distinct cell populations. **f** t-SNE plot showing the expression distribution of PRKCB in RA synovial single-cell transcriptomic data

Immune cell infiltration was quantified using the ssGSEA algorithm, and the results demonstrated significant associations between multiple immune cell populations and RA disease status (Fig. 3g). Correlation analysis further revealed that PRKCB expression was positively associated with infiltration levels of monocytes, macrophages, memory B cells, and myeloid-derived suppressor cells (MDSCs) (Fig. 4a).

Single-gene GSEA analysis demonstrated that elevated PRKCB expression was positively correlated with chemokine signaling, cytokine–cytokine receptor interaction, and hematopoietic cell lineage pathways, whereas negative correlations were observed with pathways related to circadian rhythm, dilated cardiomyopathy, and focal adhesion (Fig. 4b, c).

### Single-cell transcriptomic analysis revealed the cellular localization of PRKCB

**Fig. 5:**
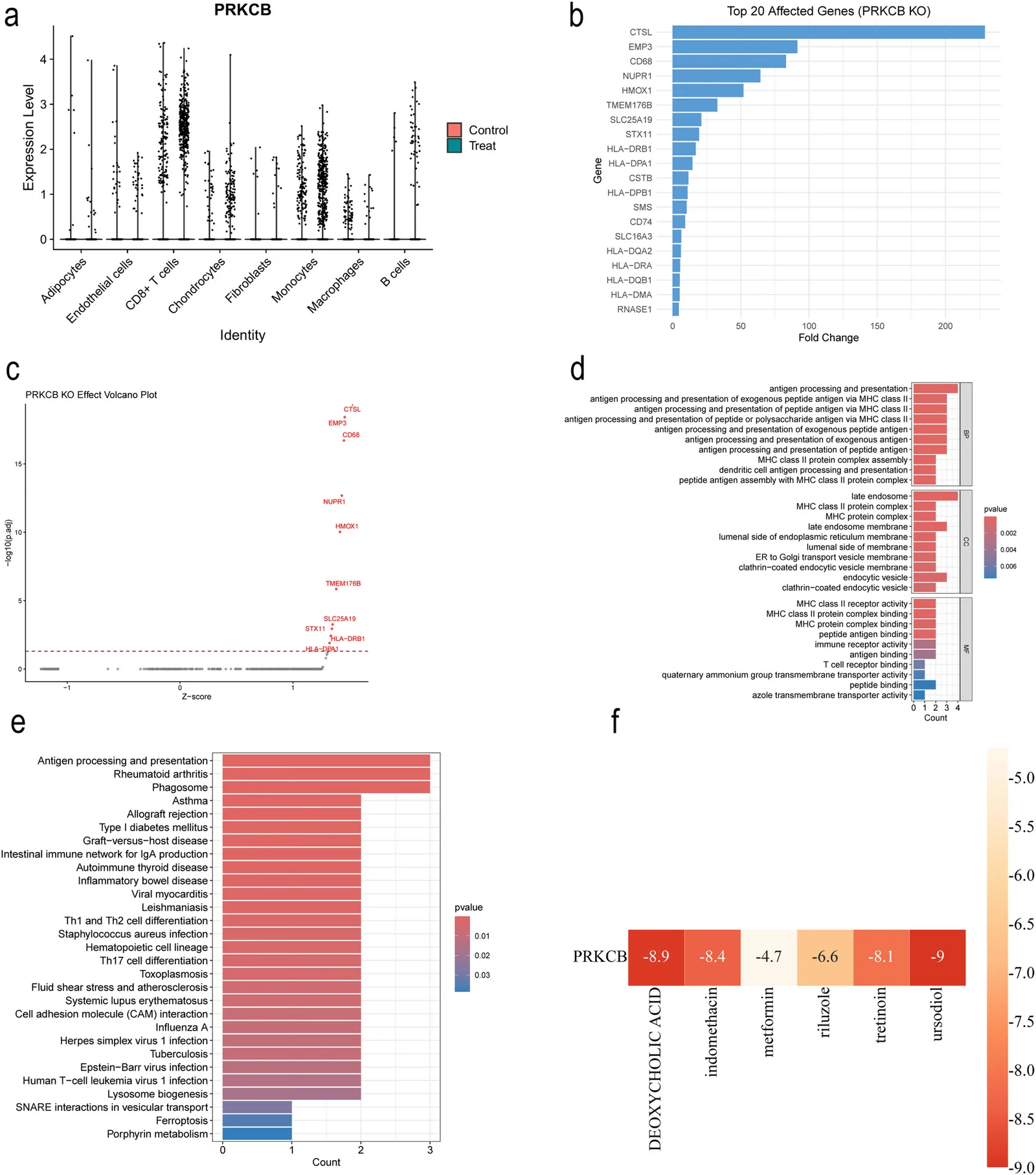
Cell-specific expression, downstream regulatory network, and candidate drug screening of PRKCB. **a** Expression distribution of PRKCB across different cell populations in RA synovial tissue. **b** Top 20 genes with the most significant expression changes following scTenifoldKnk-based virtual knockout of PRKCB. **c** Effect volcano plot of PRKCB virtual knockout. **d** GO enrichment analysis of differentially expressed genes following PRKCB knockout. **e** KEGG pathway enrichment analysis of differentially expressed genes following PRKCB knockout. **f** Heatmap of molecular docking binding energies between PRKCB and candidate drugs (unit: kcal/mol; lower values indicate stronger binding affinity)

Previous sc-eQTL analysis suggested enrichment of PRKCB expression in classical monocytes. To validate its cellular localization, single-cell transcriptomic datasets GSE200815 and GSE246416 from the GEO database, including four RA samples and five control samples, were analyzed. Following quality control and cell annotation, eight cell populations were identified, including adipocytes, endothelial cells, CD8⁺ T cells, chondrocytes, fibroblasts, monocytes, macrophages, and B cells (Fig. 4d–f). UMAP visualization and violin plot analysis consistently demonstrated relatively high PRKCB expression in the monocyte population (Fig. 5a).

### Downstream regulatory effects of PRKCB knockout simulated by scTenifoldKnk

The scTenifoldKnk framework was applied to simulate PRKCB knockout at the single-cell level to investigate its downstream regulatory network. Differential expression analysis identified CTSL as the most significantly altered downstream target gene following PRKCB knockout (Fig. 5b, c).

Functional enrichment analysis of the differentially expressed genes demonstrated significant enrichment in GO terms related to antigen processing and presentation, exogenous antigen presentation, peptide antigen presentation, late endosome, and MHC class II protein complex pathways (Fig. 5d). KEGG analysis further revealed enrichment in pathways associated with antigen processing and presentation, phagosome, asthma, Th1 and Th2 cell differentiation, and Staphylococcus aureus infection (Fig. 5e), suggesting that PRKCB may contribute to RA immune dysregulation through modulation of antigen presentation-related processes.

### Identification of candidate drugs targeting PRKCB and molecular docking analysis

Potential small-molecule compounds targeting PRKCB were screened using the DSigDB database. After exclusion of compounds with reported toxicity, six FDA-approved candidate drugs were identified, including riluzole, deoxycholic acid, ursodiol, metformin, indomethacin, and tretinoin. Molecular docking analysis demonstrated that, except for metformin, all candidate compounds exhibited binding energies below − 5 kcal/mol with PRKCB, indicating relatively stable interactions with the target protein (Fig. 5f).

**Fig. 6:**
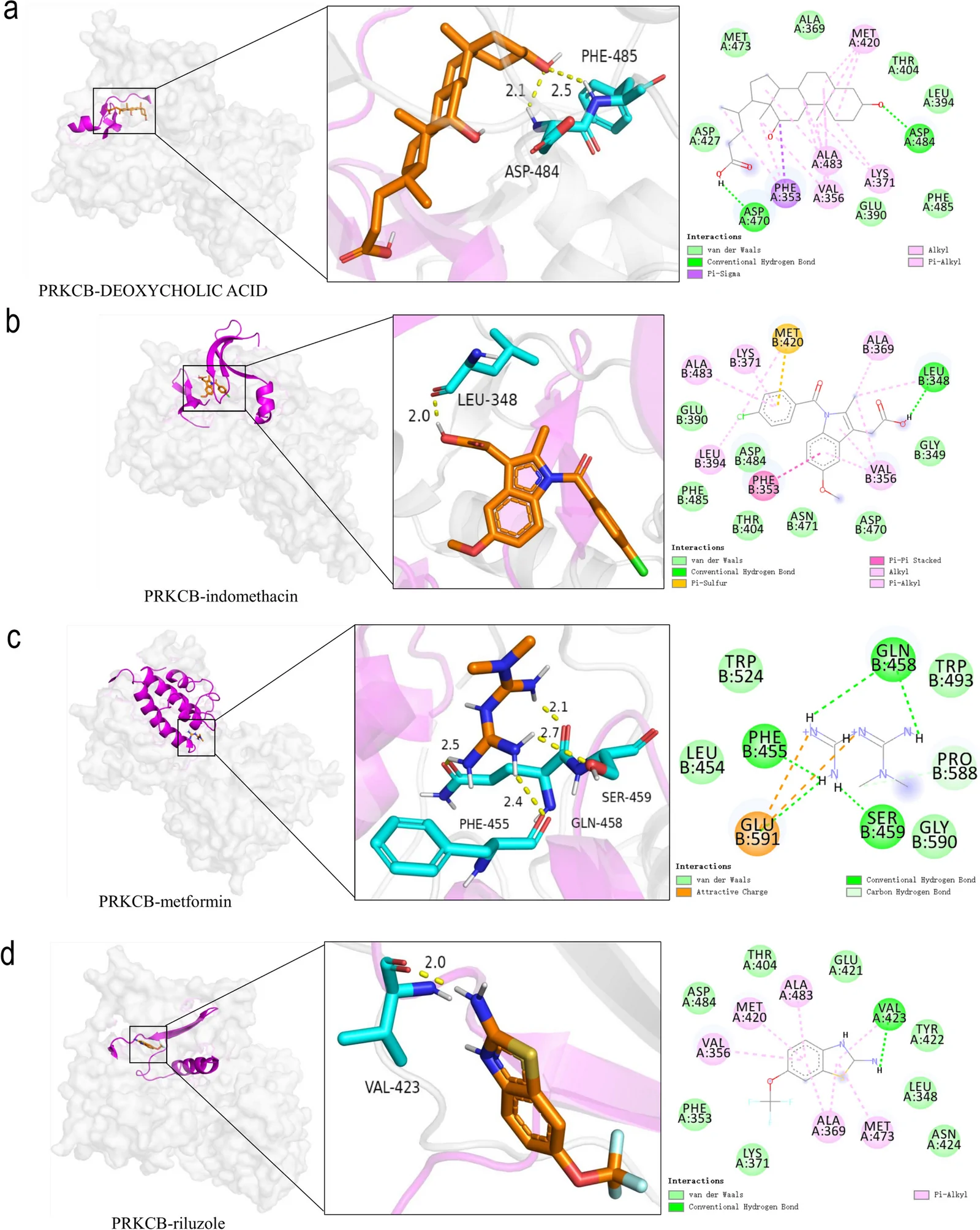
Molecular docking interaction patterns between PRKCB and candidate therapeutic compounds. **a** Binding mode of PRKCB with deoxycholic acid. **b** Binding mode of PRKCB with indomethacin. **c** Binding mode of PRKCB with metformin. **d** Binding mode of PRKCB with riluzole

**Fig. 7:**
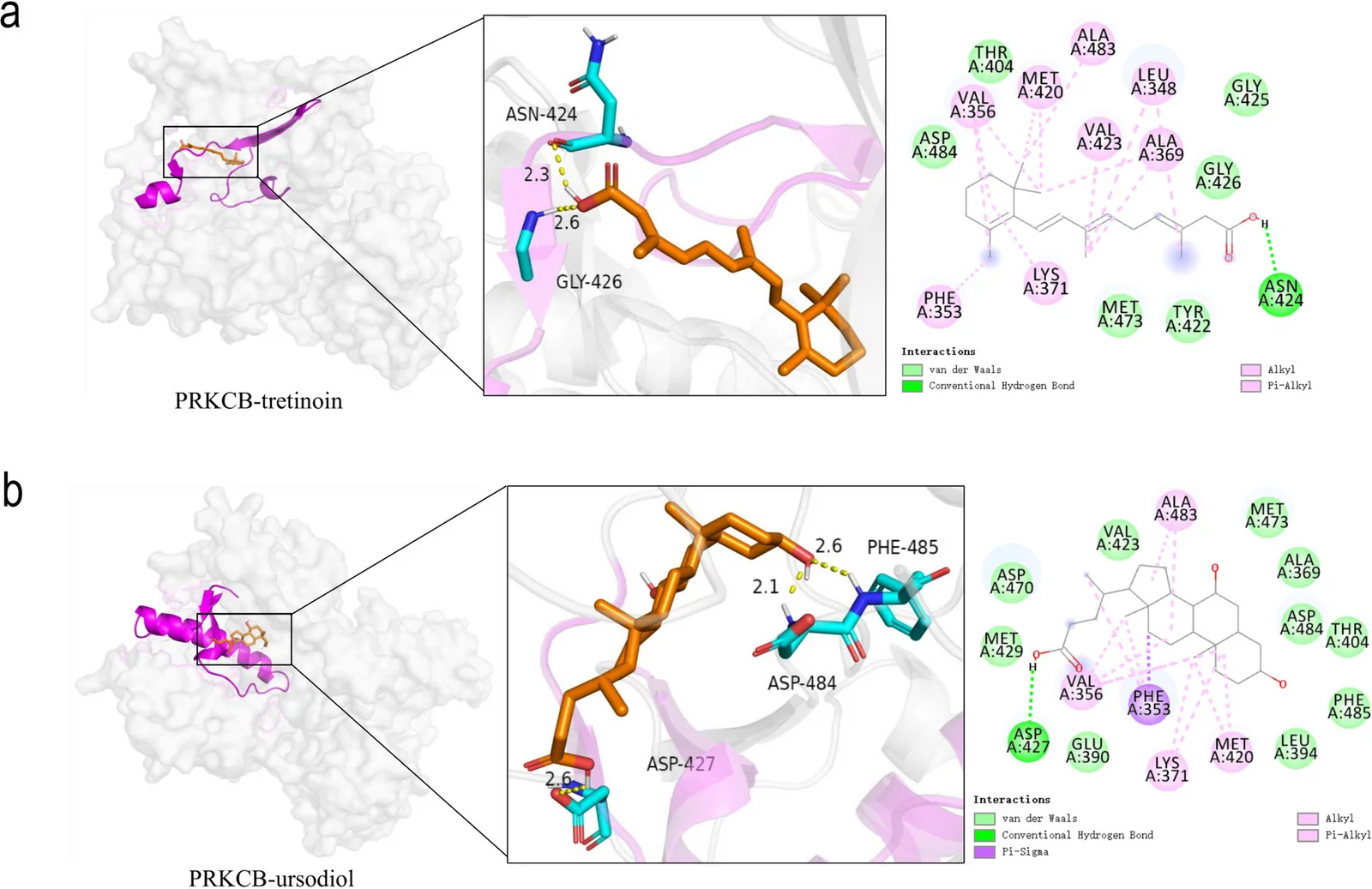
Molecular docking interaction patterns between PRKCB and candidate therapeutic compounds. **a** Binding mode of PRKCB with tretinoin. **b** Binding mode of PRKCB with ursodiol

Among these compounds, ursodiol exhibited the lowest binding energy (− 9 kcal/mol) and the strongest binding affinity toward PRKCB, and was therefore selected as the optimal candidate drug. Further docking analysis showed that all candidate compounds formed hydrogen bond interactions with key amino acid residues of the target protein (Figs. 6a–d; 7a, b).

### Molecular dynamics simulation validated the binding stability between ursodiol and PRKCB

**Fig. 8:**
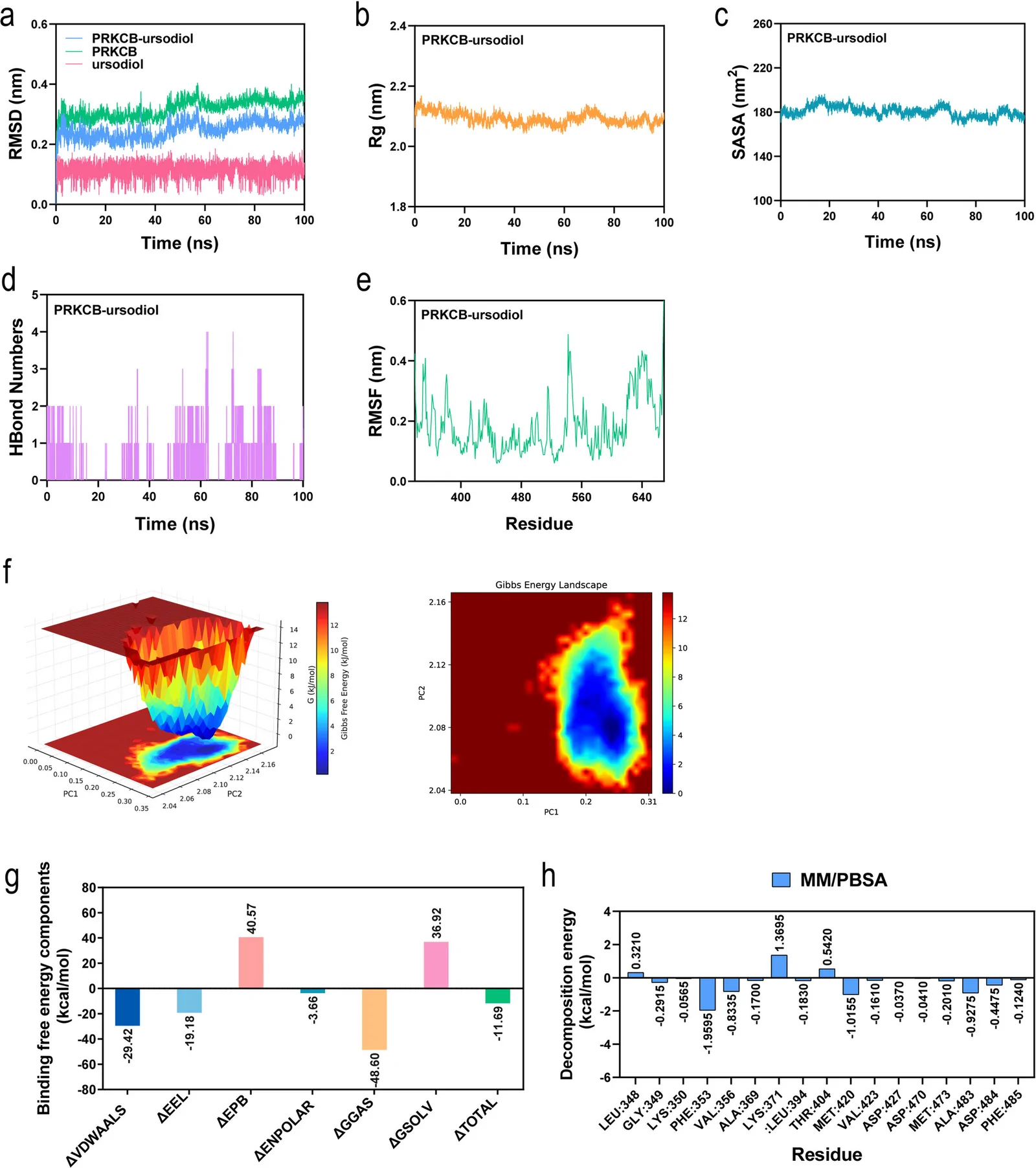
Molecular dynamics characterization of the PRKCB–ursodiol complex. **a** Time evolution of the RMSD. **b** Rg during the simulation. **c** Temporal variation in SASA. **d** Number of intermolecular hydrogen bonds formed between PRKCB and ursodiol over the simulation trajectory. **e** Residue-level RMSF of PRKCB. **f** FEL of the PRKCB–ursodiol complex. **g** Decomposition of the MM/PBSA binding free energy into individual energetic components. **h** Per-residue decomposition of the binding free energy, highlighting residues with major energetic contributions to PRKCB–ursodiol association

To further characterize the dynamic stability of the PRKCB–ursodiol complex, a 100-ns molecular dynamics (MD) simulation was performed. Root mean square deviation (RMSD) analysis showed that both apo PRKCB and the PRKCB–ursodiol complex reached relatively stable conformational states after approximately 45 ns, with RMSD values fluctuating within 0.35–0.37 nm and 0.25–0.28 nm, respectively (Fig. 8a). Free ursodiol reached equilibrium after approximately 5 ns and subsequently maintained an RMSD of 0.11–0.14 nm. These results indicate that the PRKCB–ursodiol complex remained conformationally stable during the equilibrated phase of the simulation. The radius of gyration (Rg) displayed only modest fluctuations throughout the trajectory (Fig. 8b), indicating that the overall compactness of the complex was largely maintained without substantial global structural rearrangement. Consistently, solvent-accessible surface area (SASA) analysis revealed limited fluctuations over time (Fig. 8c), suggesting relatively stable solvent exposure of the protein–ligand complex during the simulation. Hydrogen-bond analysis further demonstrated that 0–4 intermolecular hydrogen bonds were formed between ursodiol and PRKCB, with approximately one hydrogen bond being maintained during much of the simulation (Fig. 8d). This pattern suggests that hydrogen-bond interactions may contribute to stabilization of the ligand–protein interface. Root mean square fluctuation (RMSF) analysis showed relatively low residue-level fluctuations across most regions of the complex, with RMSF values predominantly ranging from approximately 0.07 to 0.40 nm (Fig. 8e), supporting limited local conformational flexibility during the simulation. Free-energy landscape (FEL) analysis identified a distinct low-energy basin near PC1 = 0.26 and PC2 = 2.135 (Fig. 8f), indicating preferential sampling of a relatively stable conformational state. MM/PBSA analysis further yielded an estimated binding free energy of − 11.69 kcal/mol for the PRKCB–ursodiol complex (Fig. 8g), consistent with an energetically favorable association under the simulated conditions. Per-residue free-energy decomposition identified Phe353, residue 420, Ala483, Val356, Asp484, and Gly349 as major contributors to the calculated ligand-binding energy (Fig. 8h). These residues may therefore contribute to stabilization of the PRKCB–ursodiol binding interface. Collectively, the RMSD, Rg, SASA, hydrogen-bond, RMSF, FEL, and MM/PBSA analyses indicate that the PRKCB–ursodiol complex maintains a relatively stable conformation and energetically favorable interaction under the simulated conditions. These structure-based findings further support ursodiol as a candidate ligand for PRKCB and warrant subsequent experimental validation of direct binding and target engagement.

### FAERS-based safety assessment of ursodiol

**Fig. 9:**
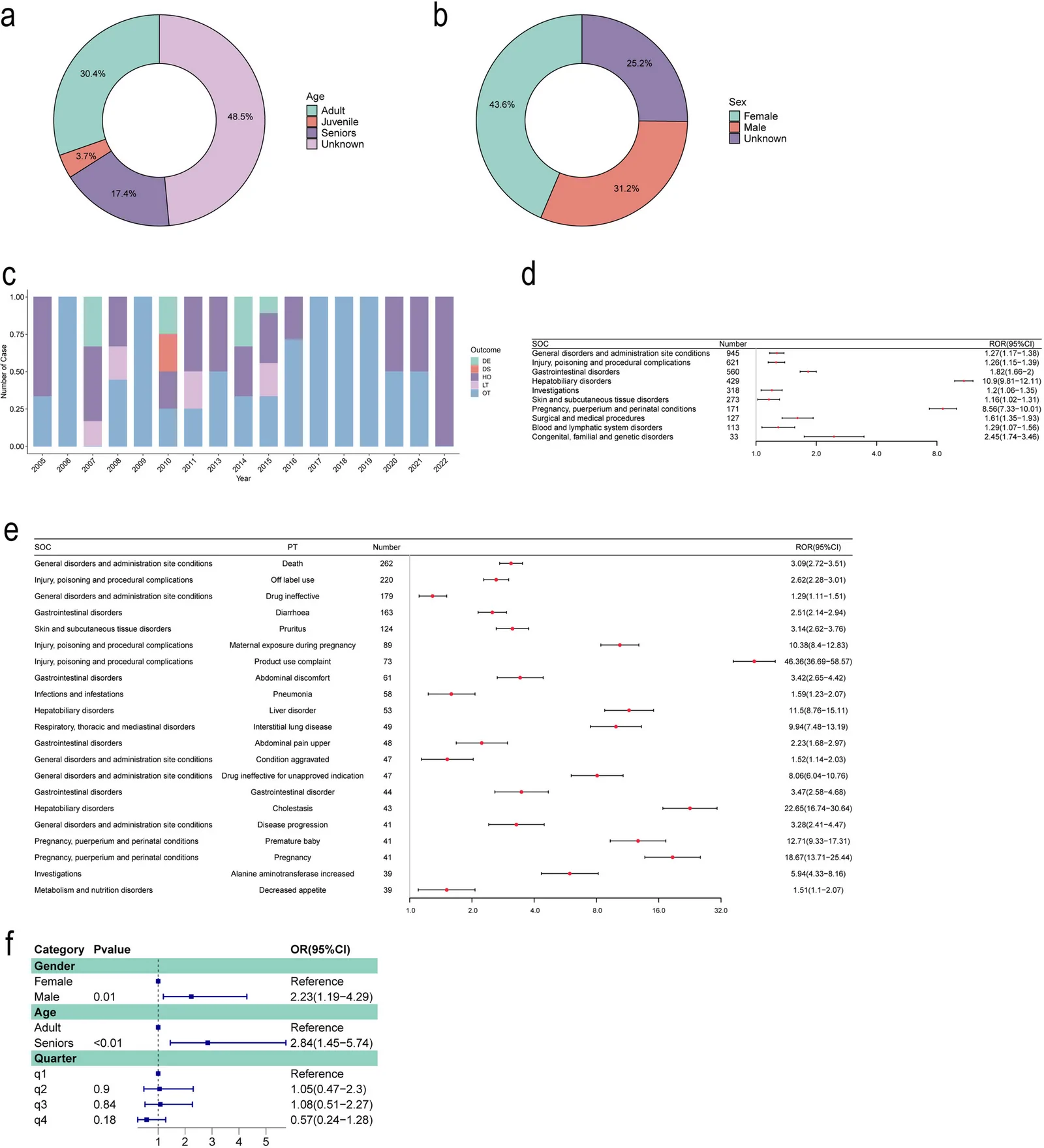
FAERS-based adverse event signal detection and safety assessment of the candidate drug ursodiol. **a**–**b** Donut plots showing demographic characteristics of adverse event reports. **a** Age distribution. **b** Sex distribution. **c** Stacked bar plot showing the annual distribution of adverse event reports. **d** Forest plot of adverse event signals at the system organ class (SOC) level. **e** Forest plot of adverse event signals at the preferred term (PT) level. **f** Forest plot of multivariable logistic regression analysis for factors associated with adverse events

Adverse event reports associated with ursodiol from the first quarter of 2004 to the second quarter of 2025 were extracted from the FAERS database. After duplicate removal, terminology standardization, and target drug screening, a total of 6214 valid adverse event reports were included for analysis. Demographic analysis indicated that ursodiol-related adverse events predominantly occurred in adults, with a higher reporting frequency in females than in males. The major clinical outcomes included hospitalization and surgical intervention (Fig. 9a–c).

SOC analysis demonstrated that adverse events were mainly associated with general disorders and administration-site conditions, injury/poisoning and procedural complications, and gastrointestinal disorders (Fig. 9d). Frequently reported adverse events included product use-related complaints, cholestasis, pregnancy exposure, and interstitial lung disease (Fig. 9e). Multivariable logistic regression analysis further demonstrated that male sex and advanced age were independent risk factors for ursodiol-associated interstitial lung disease after adjustment for age, body weight, and off-label drug use (Fig. 9f).

### In vitro validation of the PRKCB regulatory pathway

To further validate the effects of PRKCB knockdown and investigate its regulatory relationship with CTSL and NF-κB signaling, Western blot analysis was performed in the RA-related inflammatory cell model (Supplementary Fig. 2a–d). Compared with the normal control group, the RA model group exhibited significantly increased protein levels of PRKCB, CTSL, and phosphorylated NF-κB (p-NF-κB), whereas total NF-κB levels remained unchanged. These findings indicate that increased PRKCB and CTSL expression is accompanied by enhanced NF-κB phosphorylation under RA-related inflammatory conditions.

In the PRKCB knockdown experiment (Supplementary Fig. 2a, b), no significant differences in the examined protein levels were observed between the Disease + si-NC and Disease groups, indicating that the transfection procedure itself did not produce detectable nonspecific effects on these readouts. In contrast, PRKCB expression was significantly reduced in the Disease + si-PRKCB group, confirming effective PRKCB knockdown. PRKCB depletion was accompanied by significant reductions in CTSL and p-NF-κB levels, whereas total NF-κB expression remained unchanged. These results suggest that PRKCB knockdown suppresses CTSL expression and attenuates the phosphorylation-associated activation of NF-κB signaling.

To further examine the proposed downstream relationship between PRKCB and CTSL, a CTSL overexpression rescue experiment was performed (Supplementary Fig. 2c, d). Following CTSL overexpression in PRKCB-knockdown cells, CTSL protein levels were significantly restored relative to those in the Disease + si-PRKCB group. This restoration was accompanied by a significant increase in p-NF-κB levels, whereas PRKCB expression remained low. Thus, restoration of CTSL partially reversed the suppression of NF-κB signaling associated with PRKCB knockdown, providing functional evidence that CTSL may act downstream of PRKCB in this experimental context.

Collectively, these findings support a model in which PRKCB may influence NF-κB signaling, at least in part, through CTSL-associated regulation. The knockdown and rescue experiments are consistent with CTSL functioning downstream of PRKCB and suggest a potential PRKCB–CTSL–NF-κB regulatory axis in monocyte inflammatory responses. Further mechanistic studies are required to determine whether these components constitute a direct linear signaling cascade.

## Discussion

In this study, we integrated cell-type-specific sc-eQTL data, two-sample MR, multi-cohort transcriptomic analysis, WGCNA, machine learning, and single-cell transcriptomics to prioritize molecular features associated with RA. Eleven candidate targets were supported by convergent genetic and transcriptomic evidence, and their expression profiles delineated two molecular subtypes with distinct immune and pathway characteristics. Among these candidates, PRKCB was consistently prioritized by three machine-learning approaches and showed preferential expression in monocytes. Computational perturbation implicated CTSL and antigen-processing-related programs among the molecular changes associated with PRKCB perturbation, while in vitro PRKCB knockdown was accompanied by reduced CTSL expression and attenuation of NF-κB-related signaling. Structure-based analyses further identified ursodiol as a candidate compound with a favorable predicted interaction with PRKCB. Taken together, these findings support a monocyte-associated PRKCB–CTSL relationship as a testable regulatory hypothesis in RA rather than an established linear signaling mechanism. The integration of independent analytical layers strengthens the biological prioritization of this candidate pathway, while direct mechanistic and in vivo validation remains necessary.

The marked heterogeneity and polygenic regulatory characteristics of RA remain major challenges in mechanistic investigation and precision therapy. Previous studies have largely relied on single-omics association analyses, which are limited in distinguishing causal relationships from confounding effects and often lack systematic characterization of immune cell-specific regulatory networks [28–30]. In the present study, integration of single-cell eQTL and two-sample MR analyses enabled the identification of RA-associated candidate genes at the genetic level, while transcriptomic differential analysis and WGCNA further improved the biological relevance and robustness of target prioritization [31, 32]. Functional enrichment analyses demonstrated that the identified targets were primarily associated with immune regulation, cytoskeletal remodeling, and integrin binding, which are closely related to the inflammatory microenvironment and synovial tissue injury observed in RA, highlighting the value of multi-omics integration in complex autoimmune disease research.

PRKCB was identified as the core target through integrative screening using LASSO, SVM, and RF algorithms. PRKCB emerged as the most consistently prioritized candidate in the present integrative analysis. As a member of the conventional protein kinase C family, PRKCB participates in multiple immune-cell signaling programs; however, its RA-specific function remains incompletely defined. This distinction is important when interpreting our findings. The preferential expression of PRKCB in monocytes, together with its association with chemokine and cytokine-receptor pathways, supports a potential role in myeloid inflammatory states but does not establish PRKCB as a monocyte-specific pathogenic driver. Monocytes and monocyte-derived macrophages are well-established contributors to RA synovitis through cytokine production, inflammatory-cell recruitment, and tissue-remodeling processes. Thus, the convergence of the sc-eQTL and single-cell expression results provides a biologically plausible cellular context in which the function of PRKCB warrants further investigation [33–35].

The evidence supporting CTSL in RA differs from that for PRKCB. Previous studies have detected cathepsin L in RA synovial tissue and synovial fluid and have linked its expression or activity to inflammatory synovial pathology, extracellular-matrix remodeling, and processes associated with mononuclear-cell infiltration. These observations provide an established disease context for CTSL, whereas a direct PRKCB–CTSL regulatory relationship in RA has not, to our knowledge, been established [36–38]. In the present study, scTenifoldKnk-based perturbation identified CTSL among the genes most strongly altered following virtual PRKCB perturbation, and PRKCB knockdown in the cellular model was accompanied by reduced CTSL expression [39–41]. These convergent observations support a potential regulatory association between PRKCB and CTSL but do not distinguish direct transcriptional regulation from indirect effects mediated through broader inflammatory or signaling networks [42, 43]. In vitro experiments further demonstrated that PRKCB knockdown reduced CTSL expression and suppressed NF-κB pathway activation. Collectively, these findings suggest that PRKCB may contribute to monocyte-mediated inflammatory responses and tissue damage in RA through regulation of CTSL and inflammatory signaling pathways.

Drug repurposing analysis identified ursodiol as the compound with the most favorable predicted interaction with PRKCB among the candidates evaluated. Molecular docking and MD simulations provided complementary structure-based evidence that the PRKCB–ursodiol complex could adopt a relatively stable configuration under the simulated conditions. These results should, however, be interpreted as computational prioritization rather than evidence of pharmacological target engagement. Neither direct binding of ursodiol to PRKCB nor PRKCB-dependent anti-inflammatory activity of ursodiol was experimentally assessed in the present study. The FAERS analysis likewise provides a descriptive pharmacovigilance perspective on reported adverse events rather than evidence regarding efficacy in RA. Accordingly, ursodiol should presently be regarded as a candidate compound for subsequent validation. Biophysical binding assays, cellular target-engagement studies, dose–response experiments, and in vivo arthritis models will be required to determine whether the predicted PRKCB interaction has functional or therapeutic relevance.

The clinical feasibility of PRKCB-directed intervention also remains uncertain. Because PRKCB participates in signaling across multiple immune-cell contexts, systemic inhibition may not selectively affect the monocyte compartment implicated by the present analyses. The degree of target inhibition required for therapeutic activity, potential pathway redundancy, possible on-target effects in other immune populations, and the therapeutic window remain unknown. In addition, it is not yet established whether patients with increased monocyte-associated PRKCB activity constitute a molecular subgroup that would preferentially respond to PRKCB-directed intervention. Future studies should therefore combine selective pharmacological or genetic perturbation with target-engagement measurements, functional assays in primary RA-derived myeloid cells, in vivo arthritis models, and biomarker-based patient stratification before clinical translation is considered.

Several limitations should be considered. First, although integrating multiple transcriptomic datasets reduced reliance on a single discovery cohort, independent external validation and prospectively recruited multicenter RA cohorts were unavailable. Heterogeneity in patient characteristics, experimental platforms, and clinical annotation across public datasets may therefore affect generalizability, warranting validation in independent cohorts with harmonized clinical and molecular data. Second, the machine-learning analyses were intended for candidate prioritization rather than clinical prediction. To limit model-specific selection and overfitting, the feature space was restricted to 11 candidates supported by genetic, differential-expression, and co-expression evidence; cross-validation was incorporated into model optimization; and only genes consistently identified by LASSO, SVM, and Random Forest were advanced. PRKCB was further supported by single-cell localization, virtual perturbation, and experimental analyses independent of the machine-learning models. Nevertheless, internal cross-validation cannot replace external validation, and residual selection bias cannot be excluded. Third, experimental validation was confined to an in vitro inflammatory cell model. PRKCB knockdown combined with CTSL rescue provided directional molecular evidence for the proposed PRKCB–CTSL relationship, as CTSL restoration partially recovered NF-κB phosphorylation despite sustained PRKCB depletion. However, this does not establish direct PRKCB–CTSL regulation or demonstrate effects on broader RA-relevant phenotypes. Functional studies assessing cytokine secretion, migration, antigen presentation, proliferation, and apoptosis are therefore warranted. Fourth, PRKCB was perturbed using siRNA rather than CRISPR-based approaches. Although appropriate siRNA controls and CTSL rescue strengthen the observed relationship, residual target expression and potential off-target effects cannot be fully excluded. CRISPR-based perturbation would provide more stringent genetic validation. Fifth, no in vivo arthritis model was included. Thus, the current experiments cannot reproduce the complex synovial microenvironment, systemic immune interactions, or joint-level outcomes, and the biological relevance of PRKCB–CTSL signaling requires further in vivo investigation. Finally, ursodiol was prioritized computationally. Docking and molecular-dynamics simulations support a predicted structural interaction but do not demonstrate direct PRKCB binding, target engagement, pharmacological activity, or therapeutic efficacy. Similarly, FAERS provides pharmacovigilance information rather than evidence of RA efficacy. Direct target-engagement, pharmacological, and in vivo studies are therefore required before the therapeutic relevance of ursodiol can be established. Overall, the present study should be viewed as a multi-layer target-prioritization and mechanistic hypothesis-building framework rather than definitive clinical or pharmacological validation. Nevertheless, the convergence of genetic, transcriptomic, single-cell, computational, and knockdown/rescue evidence reduces reliance on any single analytical modality and provides a foundation for subsequent mechanistic and translational validation.

## Conclusion

In conclusion, integrating cell-type-specific genetic, transcriptomic, machine learning, and single-cell evidence prioritized PRKCB as a monocyte-associated candidate in RA. Computational perturbation and PRKCB knockdown/CTSL rescue supported a potential PRKCB–CTSL–NF-κB regulatory relationship, while structure-based analyses identified ursodiol as a computationally prioritized compound. These findings provide a basis for further mechanistic investigation, but independent validation, target-engagement, pharmacological, and in vivo studies are required before the therapeutic relevance of PRKCB or ursodiol can be established.

## Supplementary Information

Below is the link to the electronic supplementary material.

ESM 1 Supplementary Figure 1 (PNG 1.04 MB)

High Resolution Image (TIF 4.06 MB)

ESM 2 Supplementary Figure 2 (PNG 430 KB)

High Resolution Image (TIF 2.77 MB)

ESM3 (CSV 6.56 KB)

ESM4 (CSV 137 KB)

ESM5 (CSV 22.6 KB)

## Data Availability

Not applicable.
